# Prospective case-control study of premenopausal serum estradiol and testosterone levels and breast cancer risk

**DOI:** 10.1186/bcr2779

**Published:** 2010-11-18

**Authors:** Joanne F Dorgan, Frank Z Stanczyk, Lisa L Kahle, Louise A Brinton

**Affiliations:** 1 Fox Chase Cancer Center, 333 Cottman Avenue, Philadelphia, PA 19111, USA; 2 University of Southern California Keck School of Medicine, 1240 North Mission Road, Los Angeles, CA 90033, USA; 3 Information Management Services, 6110 Executive Boulevard, Rockville, MD 20852, USA; 4 National Cancer Institute, 6120 Executive Boulevard, Rockville, MD 20852, USA

## Abstract

**Introduction:**

Breast cancer is frequently a hormonally dependent cancer, and associations of circulating estrogens and androgens with subsequent breast cancer risk are well established in postmenopausal women. Associations of serum estrogens and androgens with breast cancer risk in premenopausal women are less well studied. The objective of this study was to determine whether estradiol and testosterone levels in serum collected before menopause are associated with subsequent breast cancer risk.

**Methods:**

We conducted a prospective case-control study of 266 participants who were registered in the Columbia, Missouri, Serum Bank and not using exogenous hormones at the time of blood collection. Each of 98 *in situ *or invasive breast cancer cases with prediagnostic serum collected before menopause was matched to two controls by age, date, menstrual cycle day, and time of day of blood collection. Estradiol and testosterone concentrations were quantified by using specific radioimmunoassays, and sex hormone-binding globulin (SHBG) was quantified with a chemiluminescent immunoassay to allow calculation of the non-SHBG bound hormone fractions. Data were analyzed by using conditional logistic regression. All tests of statistical significance were two-sided.

**Results:**

Serum testosterone was strongly and significantly associated with breast cancer risk. The relative odds (OR) for increasing quartiles of total testosterone were 1.0, 2.1 (95% confidence interval (CI) 0.9 to 4.8), 1.5 (95% CI, 0.6 to 3.4), and 3.3 (95% CI, 1.5 to 7.5, *P*_trend _= 0.006). Comparable ORs for the non-SHBG bound fraction of testosterone that is bioavailable were 1.0, 1.7 (95% CI, 0.7 to 4.2), 1.7 (95% CI, 0.7 to 4.0), and 4.2 (95% CI, 1.6 to 10.9, *P*_trend _= 0.002). Total and non-SHBG-bound estradiol were not associated with breast cancer, but extreme variation in levels across the menstrual cycle coupled with relatively small numbers, particularly for analyses stratified by cycle phase, limited the power to detect associations.

**Conclusions:**

Results suggest that premenopausal women with elevated serum testosterone levels are at an increased risk of breast cancer.

## Introduction

Most breast tumors are estrogen dependent, and postmenopausal women with elevated serum estrogens are at an increased risk of developing breast cancer [1]. Estrogens are synthesized from androgens in the premenopausal ovary and in extraovarian tissues, and postmenopausal women with elevated serum androgens are similarly at an increased risk of breast cancer [1]. Several lines of epidemiologic evidence indicate a role of premenopausal ovarian hormones in breast cancer etiology. In particular, menarche and menopause delineate the beginning and end of ovarian function, and younger age at menarche but older age at menopause are associated with an increased risk of breast cancer [2]. Thus, duration of exposure to premenopausal ovarian hormones is positively associated with risk. Evidence also is accumulating from prospective cohort studies on associations of circulating estrogens and androgens with premenopausal breast cancer risk [3-7]. However, results are not totally consistent across studies, particularly for estrogens, and additional research is needed on the long-term associations of premenopausal sex-hormone levels with breast cancer risk. Here we report results of a case-control study nested in the Columbia, MO, Serum Bank of the prospective associations of estradiol and testosterone levels in blood samples collected before menopause with subsequent breast cancer.

## Materials and methods

The Columbia, MO, Serum Bank initially was established in 1977 as part of the National Cancer Institute (NCI) Biological Markers Project to identify serum markers for breast cancer. Participants were volunteers identified primarily through the Breast Cancer Detection Demonstration Project (BCDDP) at the University of Missouri Hospital and Ellis Fischel Cancer Center in Columbia. A total of 6,915 women who initially were free of cancer, other than nonmelanoma skin cancer, donated blood to the bank on one or more occasions between December 1977 and June 1989. All women gave written informed consent before donating serum to the serum bank, and Institutional Review Boards at the National Cancer Institute and Fox Chase Cancer Center approved the research reported herein.

Serum specimens were collected, and clinical data, including age, height, weight, menstrual and reproductive histories, smoking, hormone replacement and oral contraceptive use, and family history of breast cancer, were obtained by self-report or medical-record review. Approximately 30 ml of blood was collected from each woman by using standard procedures. Blood was allowed to stand at room temperature for at least one-half hour or until it was thoroughly clotted and then refrigerated. Within 2 hours of collection, blood was centrifuged, and serum was separated and aliquoted into 1.1-ml sterile glass vials. Vials were labeled, sealed with rubber stoppers, and stored at -70°C.

Follow-up of women who donated blood to the Columbia, MO, Serum Bank was conducted in two phases. Initial follow-up continued for up to 12 years until December 1989. A questionnaire was mailed to participants annually to ascertain information on interim cancer diagnoses. Women who indicated that they had a breast biopsy or breast cancer were sent a consent form for permission to obtain medical records, including pathology reports. For cancers at sites other than the breast, date of diagnosis was ascertained. An extended follow-up of Columbia, MO, Serum Bank participants was conducted by NCI in 1999 through 2004. Cancer diagnoses were ascertained by self-report and by searching the Missouri Cancer Registry, BCDDP Cohort files, and National Death Index (NDI) Plus. Of the 6,720 women included in the extended follow-up because they had one or more vials of serum remaining, 6,131 (91%) were located as either alive or dead, 589 (9%) were not able to be located, 59 (< 1%) refused to participate, and 49 (< 1%) were too ill to participate. In total, 1,751 women (25%) were identified as deceased, with confirmation of causes of death provided via NDI.

Cases for the current study included premenopausal women in the Columbia, MO, Serum Bank who were free of cancer other than nonmelanoma skin cancer and not taking exogenous estrogens or progestins at the time they donated blood, who subsequently were diagnosed with *in situ *or invasive breast cancer that was confirmed by medical records, the Missouri Cancer Registry, or NDI. Initially, potential cases were selected if less than 1 year had elapsed since their last menses, or if they were younger than 50 years (the median age of natural menopause in the Columbia, MO, Serum Bank) at blood collection and missing data on the date of their last menses. For each of 117 potential cases that were identified, two potential controls who were alive at the age of the case diagnosis and who remained cancer free were randomly selected. Controls were matched to the case on age (± 2 years), date (± 1 year), menstrual cycle day (± 2 days, or if the potential case was missing data on the date of the last menses, a potential control with missing data on the date of her last menses was selected), and time of day (± 2 hours) of blood collection. Matching criteria were relaxed to identify potential controls in some instances (age (*n *= 6), date (*n *= 11), cycle day (*n *= 13), and time (*n *= 5)). Final determination of menopausal status for potential participants who were missing data on the date of their last menses was based on age at blood collection and serum follicle-stimulating hormone (FSH) and estradiol concentrations. Those who were younger than 44 years (the 10^th ^percentile of age at natural menopause in the Columbia, MO, Serum Bank) were presumed to be premenopausal, whereas those who were 45 to 49 years old and had an FSH greater than 25 mIU/ml and estradiol less than 25 pg/ml were considered to be postmenopausal and excluded from analysis. After exclusion of postmenopausal women and one control with serum testosterone of 102.5 ng/dl, 98 cases and 168 matched controls remained and were included in analyses.

All laboratory analyses were performed at the Reproductive Endocrine Research Laboratory, University of Southern California Keck School of Medicine. Sera from each case and the matched control were grouped together, and matched sets were randomly organized within batches. The laboratory was blinded to which samples were from cases and which were from controls. Estradiol and testosterone were quantified by specific radioimmunoassays after organic solvent extraction and Celite column-partition chromatography, as described previously [8,9]. Sex hormone-binding globulin (SHBG) was measured with a chemiluminescent immunoassay on the Immulite analyzer (Siemens Medical Solutions Diagnostics, Malvern, PA, USA) to allow calculation of bioavailable (free plus albumin bound) estradiol and testosterone [10]. FSH was similarly measured on the Immulite analyzer. The average coefficients of variation (CVs) were 8.5% for estradiol, 11.2% for testosterone, 7.0% for SHBG, and 6.9% for FSH.

### Statistical analysis

The association of serum estradiol and testosterone with breast cancer risk for matched sets was evaluated by using conditional logistic regression. Univariate associations of individual characteristics shown in Table 1 with breast cancer risk also were evaluated by using conditional logistic regression to retain the matching. Geometric mean concentrations for each hormone in cases and controls were calculated and compared by testing the statistical significance of the trend of the log-transformed variable entered as a continuous term in conditional logistic regression models. Women were stratified into quartiles of hormone concentration, and a set of categoric indicator variables was included in conditional logistic regression models to estimate odds ratios (ORs). Models were fit by using natural log-transformed hormone concentrations to test for trend. Adjusted models included terms for height (continuous), body mass index (BMI, continuous), age at menarche (continuous), number of term pregnancies (continuous), smoking (never, former, or current), and history of breast cancer in a first-degree relative (yes or no). Although controls were matched to cases on age and menstrual cycle day at blood collection, to adjust for possible residual confounding, age (continuous) was included in all models, and menstrual cycle day (cubic spline) was included in models for estradiol. Interactions were evaluated by testing the statistical significance of cross-product terms included in models with main effects. Because estradiol is secreted primarily by ovarian follicles during the follicular phase of the menstrual cycle but by the corpus luteum in the luteal phase, we also conducted estradiol analyses stratified by cycle phase at blood collection (follicular, 0 to 13 days since start of last menses; luteal, 14 to 33 days since start of last menses). Women whose blood was collected more than 33 days after the last menses or who were missing data on time since the last menses were excluded. Women were categorized into cycle phase-specific tertiles of estradiol for these analyses, because small numbers did not allow finer gradation. All tests of statistical significance were two-sided, and a cut-off of *P *less than 0.05 was used to determine statistical significance. All analyses were performed by using SAS 9.2 (Cary, NC, USA) and STATA10.1 (College Station, TX, USA).

**Table 1:** Participant characteristics at blood collection

Characteristic	Cases (*n *= 98)	Controls (*n *= 168)	
Continuous variables	**Mean ± SD**		***P***^**a**^
	
Age, years	44.7 ± 4.8	44.6 ± 4.5	0.10
Height, cm	163.4 ± 6.0	163.5 ± 5.8	0.95
BMI, kg/m^2^	25.7 ± 5.1	25.2 ± 4.8	0.35
Menarche, years	12.5 ± 1.3	12.8 ± 1.4	0.08
Age at first pregnancy, years^b^	22.2 ± 4.3	22.1 ± 4.1	0.92
Number of term pregnancies	2.7 ± 1.6	2.9 ± 1.7	0.50
			
Categoric variables	**Frequency, number (%)**		***P***^**a**^
	
Nulliparous	9 (9.2%)	13 (7.7%)	0.82
Smoking status			0.50
Never	61 (62.2%)	103 (61.3%)	
Former	19 (19.4%)	26 (15.5%)	
Current	18 (18.4%)	39 (23.2%)	
History of breast cancer in first-degree relative	17 (17.4%)	14 (8.3%)	0.03
Menstrual cycle day at blood collection			0.45
Days 0-13	35 (35.7%)	68 (40.5%)	
Days 14-33	40 (40.8%)	68 (40.5%)	
Days ≥34	9 (9.2%)	13 (7.7%)	
Unknown	14 (14.3%)	19 (11.3%)	

BMI, body mass index; SD, standard deviation. ^a^*P *values (two-sided) were calculated by using conditional logistic regression with a Wald test. ^b^Includes 89 parous cases and 155 parous controls.

## Results

Participant characteristics are summarized in Table 1. All women were premenopausal and not using oral contraceptives or other estrogens at the time of blood collection. All subjects were white. Cases' and controls' ages averaged 44.7 years (range, 31.4 to 56.1 years). Cases did not differ from controls in terms of anthropometric or reproductive characteristics, except cases experienced menarche at a slightly younger age compared with controls (12.5 versus 12.8 years; *P *= 0.08). More cases had a history of breast cancer in a first-degree relative compared with controls (17.4% versus 8.3%; *P *= 0.03). Data on date of last menses were missing for 14.3% of cases and 11.3% of controls, and 9.2% of cases and 7.7% of controls had blood collected 34 or more days after their last menses. The median number of days from last menses at blood collection for these participants was 52 days (range, 35 to 81 days). Cases' mean (± SD) age at breast cancer diagnosis was 58.7 ± 8.4 years (range, 37.7 to 74.0 years). The mean (± SD) time from blood collection to diagnosis was 14.0 ± 6.6 years. Eighty-six tumors (88%) were invasive.

As shown in Table 2 cases had statistically significantly higher geometric mean total testosterone (28.1 versus 24.7 ng/dl; *P *= 0.005) and bioavailable testosterone (11.8 versus 10.1 ng/dl; *P *= 0.002) concentrations than did controls. Total and bioavailable estradiol concentrations did not differ between the groups. Breast cancer risk also was not associated with total or bioavailable estradiol levels overall or in analyses stratified by menstrual cycle phase (Table 3). In contrast, total and bioavailable testosterone levels were strongly and significantly associated with risk (Table 4). The association was strongest for bioavailable testosterone in which the multivariable adjusted odds ratios (OR) for increasing quartiles were 1.0, 1.7, 1.7, and 4.2 (95% CI, 1.6 to 10.9; *P*_trend _= 0.002). Results were not substantially different when analysis was restricted to women who were within 33 days of last menses at the time of blood collection.

**Table 2:** Geometric mean (95% confidence interval) hormone concentrations in prediagnostic serum from cases^a ^and matched controls

	Cases			Controls			
			
	*n*	Mean	95% CI	*n*	Mean	95% CI	***P***^b^
Estradiol, pg/ml	98	85.8	72.5-101.5	168	80.3	70.5-91.6	0.62
Bioavailable estradiol, pg/ml	98	49.9	42.1-59.0	168	45.7	40.2-51.9	0.46
Testosterone, ng/dl	98	28.1	26.2-30.1	168	24.7	23.3-26.1	0.005
Bioavailable testosterone, ng/dl	98	11.8	10.8-12.8	168	10.1	9.5-10.7	0.002
SHBG, nmol/L	98	55.2	50.4-60.4	168	57.7	53.7-62.0	0.34

All variables are continuous. CI, confidence interval; SHBG, sex hormone-binding globulin. ^a^Mean (± SD) time from blood collection to diagnosis was 14.0 ± 6.6 years. ^b^*P *values (two-sided) were calculated by using conditional logistic regression with log-transformed hormone and SHBG levels entered as a linear term to test for trend by using a Wald test.

**Table 3:** Odds ratios (ORs) and confidence intervals (CIs) of breast Cancer by prediagnostic serum estradiol concentration stratified by menstrual-cycle phase ^a^

Hormone	Number of cases/number of controls	Unadjusted			Adjusted		
			
		OR	(95% CI)	***P***^**b**^	OR	(95% CI)	***P***^**b**^
**All participants**							
Estradiol				0.62			0.48
5-54 pg/ml	22/45	1.0			1.0		
55-94 pg/ml	28/37	1.7	(0.8 to 3.6)		1.9	(0.8 to 4.6)	
95-138 pg/ml	24/43	1.2	(0.6 to 2.5)		1.1	(0.5 to 2.5)	
139-497 pg/ml	24/43	1.1	(0.5 to 2.2)		1.4	(0.6 to 3.4)	
							
Bioavailable estradiol				0.46			0.38
1.5-31.3 pg/ml	23/43	1.0			1.0		
31.6-54.9 pg/ml	25/42	1.2	(0.6 to 2.5)		1.2	(0.5 to 2.8)	
55.0-79.2 pg/ml	26/41	1.2	(0.6 to 2.6)		1.2	(0.5 to 2.8)	
79.3-302.4 pg/ml	24/42	1.1	(0.5 to 2.2)		1.3	(0.5 to 3.1)	
							
**Follicular**^c^							
Estradiol				0.52			0.69
5-54 pg/ml	10/22	1.0			1.0		
56-108 pg/ml	13/21	1.5	(0.5 to 4.3)		2.4	(0.6 to 10.6)	
110-480 pg/ml	12/21	1.4	(0.4 to 4.2)		1.4	(0.3 to 6.5)	
							
Bioavailable estradiol				0.51			0.70
1.5-31.7 pg/ml	12/21	1.0			1.0		
32.2-62.9 pg/ml	8/25	0.6	(0.2 to 1.9)		0.7	(0.1 to 3.1)	
63.5-264.2 pg/ml	15/18	1.8	(0.6 to 5.8)		2.0	(0.5 to 8.3)	
							
**Luteal^d^**							
Estradiol				0.49			0.62
6-78 pg/ml	10/24	1.0			1.0		
80-114 pg/ml	16/18	2.0	(0.7 to 5.8)		2.3	(0.6 to 9.2)	
115-299 pg/ml	11/23	1.2	(0.4 to 3.2)		1.3	(0.4 to 4.3)	
							
Bioavailable estradiol				0.29			0.51
3.5-48.4 pg/ml	9/25	1.0			1.0		
48.8-67.9 pg/ml	15/19	2.1	(0.7 to 6.0)		2.5	(0.6 to 10.5)	
68.2-184.6 pg/ml	13/21	1.6	(0.6 to 4.4)		1.8	(0.6 to 5.8)	

^a^Cases and controls were matched on age, date, hour, and days since last menses at blood collection. All adjusted models include terms for age and menstrual-cycle day at blood collection, height, BMI, age at menarche, number of term pregnancies, smoking status, and history of breast cancer in first-degree relative. Hormone cut points define quartiles or menstrual-cycle phase-specific tertiles estimated from all participants. ^b^*P *values (two-sided) were calculated by using conditional logistic regression, with log-transformed hormone concentrations entered as a linear term to test for trend by using a Wald test. ^c^The case and matched controls were in the follicular phase of the menstrual cycle at blood collection (0 to 13 days since last menses). **^d^**The case and matched controls were in the luteal phase of the menstrual cycle at blood collection (14 to 33 days since last menses).

**Table 4:** Odds ratios (ORs) and confidence intervals (CIs) of breast cancer by prediagnostic serum testosterone and SHBG concentration^a^

Hormone	Number of cases/Number of controls	Unadjusted			Adjusted		
			
		OR	(95% CI)	***P***^**b**^	OR	(95% CI)	***P***^**b**^
**All participants**							
Testosterone				0.005			0.006
9.4-20.0 ng/dl	17/49	1.0			1.0		
20.1-26.9 ng/dl	27/40	1.9	(0.9 to 4.2)		2.1	(0.9 to 4.8)	
27.0-34.3 ng/dl	22/45	1.3	(0.6 to 2.9)		1.5	(0.6 to 3.4)	
34.4-65.5 ng/dl	32/34	2.9	(1.4 to 6.3)		3.3	(1.5 to 7.5)	
							
Bioavailable testosterone				0.002			0.002
3.0-8.0 ng/dl	19/46	1.0			1.0		
8.1-10.8 ng/dl	21/46	1.4	(0.6 to 3.1)		1.7	(0.7 to 4.2)	
10.9-13.8 ng/dl	24/45	1.4	(0.6 to 3.0)		1.7	(0.7 to 4.0)	
14.0-30.4 ng/dl	34/31	3.2	(1.4 to 7.1)		4.2	(1.6 to 10.9)	
							
SHBG				0.34			0.48
11.3-42.2 nmol/L	25/41	1.0			1.0		
42.5-57.9 nmol/L	27/40	1.0	(0.5 to 2.0)		1.3	(0.6 to 2.8)	
58.3-77.6 nmol/L	25/42	0.9	(0.5 to 1.9)		1.1	(0.5 to 2.8)	
78.1-198.0 nmol/L	21/45	0.7	(0.3 to 1.5)		0.8	(0.3 to 2.0)	

^a^Cases and controls were matched on age, date, hour, and days since last menses at blood collection. All adjusted models include terms for age at blood collection, height, BMI, age at menarche, number of term pregnancies, smoking status, and history of breast cancer in a first-degree relative. Hormone cut-points define quartiles estimated from all participants.

^b^*P *values (two-sided) were calculated by using conditional logistic regression with log-transformed hormone and SHBG concentrations entered as a linear term to test for trend by using a Wald test.

When we restricted analysis to invasive breast cancer (*n *= 86), results did not differ materially from those for all cases. However, a pattern of increasing risk with increasing total estradiol concentration in the follicular phase of the menstrual cycle emerged that was not apparent previously. Adjusted ORs for invasive breast cancer with increasing tertiles of follicular-phase total estradiol were 1.0, 1.8, and 2.0 (95% CI, 0.4 to 10.3), but the test for trend remained nonsignificant (*P*_trend _= 0.40). In contrast, associations of follicular-phase bioavailable estradiol with invasive cancer remained erratic, with adjusted ORs decreasing to 0.7 (95% CI, 0.1 to 3.8) and then increasing to 3.2 (95% CI, 0.6 to 16.5) across increasing tertiles of concentration.

We previously showed a strong positive association of serum müllerian-inhibiting substance (MIS) with breast cancer risk. Adjustment for MIS attenuated only slightly the associations of total and non-SHBG-bound testosterone with breast cancer risk. In analysis adjusted for MIS in addition to other covariates, ORs for increasing quartiles of total testosterone were 1.0, 2.1, 1.2, and 3.0 (95% CI, 1.2 to 7.3; *P*_trend _= 0.03). Comparable ORs for non-SHBG-bound testosterone were 1.0, 1.6, 1.3, and 3.6 (95% CI, 1.3 to 9.9; *P*_trend _= 0.02). Adjustment for MIS did not materially change results for total estradiol or non-SHBG-bound estradiol. Tests for interaction between MIS and total or bioavailable estradiol and testosterone in relation to breast cancer risk were not statistically significant.

Because we did not have data on eventual age at menopause, we could not perform an analysis stratified by menopausal status at diagnosis. We, therefore, performed analysis limited to the 67 cases who were 55 years or older at diagnosis and their matched controls as a proxy for postmenopausal status at diagnosis. Serum total and bioavailable testosterone remained significantly associated with breast cancer risk in these analyses. Covariate adjusted ORs for increasing quartiles of total testosterone were 1.0, 2.4, 1.6, and 4.5 (95% CI, 1.6 to 13.0; *P*_trend _= 0.009) and for bioavailable testosterone were 1.0, 2.1, 2.1, and 7.1 (95% CI, 2.1 to 24.3; *P*_trend _= 0.002). Few cases (*n *= 31) were younger than 55 years old at diagnosis, and results are not shown for this subgroup.

The time from blood collection to diagnosis ranged from 2 months to 23 years. To evaluate the potential for reverse causality as an explanation for the observed association between serum testosterone and breast cancer risk, we repeated analyses restricted to cases whose blood was collected longer in time before diagnosis. Results were similar to those shown in Table 4 for analysis restricted to 83 cases diagnosed at least 5 years after blood collection and when restricted to 73 cases diagnosed at least 10 years after blood collection. In analysis restricted to cases diagnosed at least 10 years after blood collection, ORs for increasing quartiles of total testosterone were 1.0, 1.7, 1.6, and 3.7 (95% CI, 1.3 to 10.3; *P*_trend _= 0.02). For non-SHBG-bound testosterone, comparable ORs were 1.0, 2.1, 2.3, and 6.2 (95% CI, 1.9 to 20.7; *P*_trend _= 0.003).

## Discussion

The positive associations that we observed in the Columbia, MO, cohort between premenopausal serum testosterone levels and breast cancer risk are consistent with those reported previously in prospective studies. In the study of Hormones and Diet in the Etiology of Breast Tumors (ORDET) [5], the breast cancer OR for premenopausal women in the highest versus lowest tertile of free testosterone was 2.8 (95% CI, 1.1 to 7.3). The excess risk of breast cancer associated with elevated testosterone levels was less in the European Prospective Investigation into Cancer and Nutrition (EPIC) but remained statistically significant; the OR for women in the highest versus lowest quartile of total testosterone was 1.7 (95% CI, 1.2 to 2.6) [4]. Premenopausal testosterone levels were not significantly associated with all breast cancers in the Nurses Health Study II (NHS-II) [3]. However, testosterone in plasma collected during the luteal phase of the menstrual cycle was significantly associated with invasive and estrogen/progesterone-receptor positive breast cancer; ORs for women in the highest versus lowest quartiles of total testosterone were 2.0 (95% CI, 1.1 to 3.6) and 2.9 (95% CI, 1.4 to 6.0), respectively, and associations were similar for free testosterone. Premenopausal testosterone levels were not associated with breast cancer risk in the Guernsey, UK, cohort [6] or in a small study based on the Washington Co., Maryland, cohort [11]. Overall, however, our data add to the growing body of evidence that supports an association between premenopausal testosterone levels and breast cancer risk.

The association of premenopausal serum estradiol with breast cancer risk is less clear. Similar to our results, premenopausal serum estradiol was not associated with risk in EPIC [4] or in the Washington Co., MD, cohort [11,12]. In contrast, statistically significant and positive associations between premenopausal serum estradiol levels were reported in other cohorts [3,6,7]. However, the association was limited to the midcycle phase of the menstrual cycle in the Guernsey cohort [6] and to the follicular phase in the NHS-II [3]. The association of follicular-phase estradiol with risk in the NHS-II was stronger for invasive breast cancer. Although we also observed a pattern of increasing risk with increasing follicular-phase estradiol levels in analyses restricted to invasive breast cancer cases, the trend remained nonsignificant. Serum estradiol concentrations vary by more than 100-fold over the duration of the menstrual cycle [13]; thus, difficulties adequately controlling for cycle day when blood was collected may explain differences in results across studies, including the null results that we and others have observed.

Elevated testosterone levels in serum collected before menopause remained associated with an increased risk of breast cancer in analyses limited to cases who were 55 years and older and presumably postmenopausal at diagnosis. We previously reported a significant increased risk of breast cancer among postmenopausal women with elevated testosterone levels in the Columbia, MO, cohort [14], and a positive association of testosterone with breast cancer risk in postmenopausal women is now well established [1]. In contrast to our study, almost all cases included in the analysis of premenopausal serum testosterone and breast cancer risk based on the NHS-II were premenopausal at diagnosis [3]. Similarly, the vast majority of cases included in the analysis from EPIC were diagnosed before 55 years of age [4], and the oldest case in the analysis from ORDET was 58 years old at diagnosis [5]. Our finding that the association of premenopausal levels of serum testosterone with breast cancer persists after menopause extends these earlier findings and suggests possible longer-term effects of premenopausal hormone levels on breast cancer risk. Alternatively, women with higher testosterone levels before menopause may continue after menopause to have higher levels that confer increased risk. Women's testosterone levels decline as a function of age from 20 to 50 years [15-18]. However, the ovaries continue to secrete testosterone after menopause [15,19], and most studies indicate that serum testosterone levels do not decrease during the menopausal transition [15,20] or decrease only slightly [21]. We only measured testosterone in a single blood sample collected before menopause. However, the limited data available suggest that levels track over time in the same women. The intraclass correlation coefficient of testosterone levels in plasma samples collected from premenopausal women 3 years apart was 0.73 in one study [22]. Furthermore, within-person correlations of testosterone in blood samples collected 6 years apart during the menopausal transition was 0.25 [23]. Thus, the association that we observed between higher premenopausal testosterone levels and increased postmenopausal breast cancer risk could be related to early effects of testosterone on tumor development, or higher premenopausal levels of testosterone could predict higher levels later in life that are associated with increased risk.

The association of serum testosterone with breast cancer has been reported to be stronger for estrogen receptor-positive versus -negative tumors [3,24,25], and one mechanism by which testosterone is hypothesized to increase breast cancer risk is through conversion to estradiol. Breast tissue estradiol is derived from direct uptake from the circulation and by local synthesis from precursors, such as testosterone [26]. Aromatase, which catalyzes the conversion of androgens to estrogens, is expressed by adipose tissue fibroblasts in normal breast and contributes to local estrogen production [27]. Breast tumors also can produce high levels of aromatase, and tumor-derived factors can stimulate local aromatase expression by adipose fibroblasts [27]. Although considerable interindividual variation exists, breast tumor estrogen levels are higher than those in normal breast tissue and than plasma levels in postmenopausal women [28]. Treatment with aromatase inhibitors dramatically lowers estrogen levels systemically and in breast tissue [29]. Thus, even though uptake from the circulation [30] and other pathways (for example, sulfatase) [31] may also contribute to elevated levels of estrogens in breast tumors, metabolism of androgens, including testosterone, by aromatase is an important source.

We previously observed a positive association of serum MIS with breast cancer risk in our study participants [32]. Because MIS participates in regulation of ovarian folliculogenesis [33], we speculated that defects in ovarian follicle development may contribute to elevated hormones associated with breast cancer risk. However, adjustment for MIS in the current analysis attenuated associations of total and non-SHBG-bound testosterone with breast cancer only slightly, and women in the highest quartiles remained at significantly increased risk. Thus, additional ovarian factors as well as other sources, such as the adrenals through direct secretion or secretion of testosterone precursors, may also contribute to elevated testosterone in women in whom breast cancer develops.

Our study had several strengths. Most notably, hormones were measured in serum that was collected many years before the cases' breast cancer diagnoses, and cohort follow-up was 91% complete. It also had several potential limitations. Menstrual cycle day of blood collection was calculated as days since the last menses, even though days until the next menses more accurately estimate the cycle day. This is particularly of concern for estradiol, because of its large fluctuations across the menstrual cycle and may have contributed to lack of an apparent association of estradiol with breast cancer risk. Our relatively small sample size, particularly for stratified analyses, also limited the power to detect associations.

## Conclusions

Results suggest that premenopausal women with elevated serum total and non-SHBG-bound testosterone levels are at an increased risk of subsequent breast cancer.

## Abbreviations

BCDDP: Breast Cancer Detection Demonstration Project; BMI: body mass index; CI: confidence interval; DHEA: dehydroepiandrosterone; EPIC: European Prospective Investigation into Cancer and Nutrition; FSH: follicle-stimulating hormone; MIS: müllerian-inhibiting substance; MO: Missouri; NCI: National Cancer Institute; NDI: National Death Index; NHS-II: Nurses' Health Study II; OR: odds ratio; ORDET: Hormones and Diet in the Etiology of Breast Tumors; SHBG: sex hormone-binding globulin.

## Competing interests

The authors declare that they have no competing interests.

## Authors' contributions

JFD participated in the design of the study, performed the statistical analysis, and contributed to manuscript preparation. FZS performed serum hormone analyses and contributed to manuscript preparation. LLK participated in the design of the study and case-control identification. LAB participated in the design of the study and manuscript preparation. All authors read and approved the final manuscript.
